# General health factors may be a barrier to effective non-surgical multidisciplinary rehabilitation of common orthopaedic conditions in tertiary care settings

**DOI:** 10.1186/s12891-018-2265-6

**Published:** 2018-09-27

**Authors:** Shaun O’Leary, Michelle Cottrell, Maree Raymer, David Smith, Asaduzzaman Khan

**Affiliations:** 1 School of Health and Rehabilitation Sciences, University of Queensland, Brisbane, Australia; 2 Physiotherapy Department, Royal Brisbane and Women’s Hospital, Brisbane, Australia; 3 Physiotherapy Department, Ipswich Hospital, Ipswich, Australia

**Keywords:** Musculoskeletal, Shoulder impingement, Knee osteoarthritis, Low back pain, Predictors, Non-surgical management

## Abstract

**Background:**

To explore patient characteristics predictive of a poor response to multidisciplinary non-surgical rehabilitation of three common orthopaedic conditions within a tertiary care service.

**Methods:**

A retrospective audit of medical records of patients who had undergone multidisciplinary non-surgical management of their knee osteoarthritis (KOA, *n* = 190), shoulder impingement syndrome (SIS, *n* = 199), or low back pain (LBP, *n* = 242) within a multisite tertiary care service was undertaken. Standardised clinical measures recorded by the service at the initial consultation were examined using a base binary logistic regression model to determine their relationship with a poor response to management (ie. not achieving a minimal clinically important improvement in the condition disability measure pre-post management).

**Results:**

Factors predictive of a poor response following non-surgical management included;; higher levels of anxiety (OR 1.11, *P* < 0.02) and lower functional score (OR 0.76, *P* < 0.04) for KOA, higher number of comorbidities (OR 1.16, *P* < 0.03) for SIS, and coexisting cervical or thorax pain (OR 2.1, *P* = 0.04) and lower pain self-efficacy (OR 0.98, *P* = 0.02) for LBP.

**Conclusions:**

General health issues may present a barrier to achieving favourable outcomes in response to multidisciplinary non-surgical rehabilitation for the management of common orthopaedic conditions in a tertiary care setting. Clinicians may need to consider these broader patient issues when designing management strategies for patients with these conditions.

## Background

Chronic musculoskeletal conditions such as knee osteoarthritis (KOA), shoulder impingement syndrome (SIS) and low back pain (LBP) are one of the largest causes of disability in the community [1]. Often non-surgical management is the first line of care for these conditions. While non-surgical multidisciplinary management may benefit some patients with these conditions [2–4], modest outcomes from intervention trials indicate that many patients do not benefit. It would be clinically and economically advantageous to be able to identify characteristics of patients most likely to have a poor response to multidisciplinary non-surgical management. However, identifying patient characteristics of those at risk of not responding to this pathway of care is still a work in progress.

Studies identifying characteristics of patients potentially at risk of poor recovery from common musculoskeletal condition such as KOA, SIS, and LBP are emerging. However many studies only report factors associated with poorer outcome following surgery [5–13] or natural recovery [14]. Only a few studies have specifically investigated risk factors in the context of response to non-surgical rehabilitation [3, 15–19]. For example factors shown to be associated with poor response to physical interventions for KOA include patellofemoral pain, anterior cruciate ligament laxity, and greater height [15]. Similarly, symptom severity, poorer patient expectations, low self-efficacy, compensable claims history, and co-morbidities have been reported as some of the potential risk factors for a poor response to non-surgical rehabilitation in shoulder conditions [3, 16–18]. Pain severity and psychological factors such as catastrophizing or depressed mood have also been identified as predictive of a poor response to non-surgical management of LBP [19].

There are also some methodological limitations in this field of research investigating risk factors for a poor response to non-surgical rehabilitation. For example studies may only focus on a particular domain such as clinical severity [17], physical findings [20], psychosocial factors [21], or adherence to recommended management strategies [22–25]. Furthermore some studies limit their investigation to the response to unimodal interventions (eg. manipulation) only [26, 27] or in response to treatment from a single professional discipline (eg. physiotherapy) [16, 19]. A final, but particularly relevant limitation is that studies may be confined to a primary care setting [14, 19]. This may have implications when findings are extrapolated to those patients managed in secondary or tertiary care settings where both the patient demographic and the specifics of the non-surgical service and interventions may be very different. Clearly it is challenging for any single predictive study to account for all potential risk factors, patient populations, intervention types, or variations in service settings.

The aim of this study is to further explore potential predictors of a poor response to the non-surgical management of KOA, SIS, and LBP, but is focused to a multidisciplinary intervention conducted within a tertiary care setting. It describes a retrospective audit of medical records from a representative sample of patients with known standardised service relevant clinical outcome measures and assessment items, taken over a course of non-surgical multidisciplinary rehabilitation for their orthopaedic condition within a multisite tertiary healthcare service.

## Methods

### Study design

A retrospective medical record audit was undertaken at seven public hospitals in Queensland, Australia. All cases included in the audit were patients of a standardised state-wide physiotherapy-led orthopaedic tertiary service within Queensland Health Hospital facilities between July 2008 and June 2010. These patients had been selected from specialist orthopaedic outpatient waiting lists following a triage process to undergo a course of non-surgical multidisciplinary (physiotherapy, occupational therapy, dietetics, and/or psychology) management for their KOA, SIS, or LBP. Each patient’s management (duration of management period, disciplines consulted) had been pragmatically based on the initial examination findings of the triaging service team leader (who was a physiotherapist) and the clinical discretion of the involved discipline-specific treatment providers.

This project received multisite ethical approval from the Institutional Medical Research Ethics Committee (HREC/10/QRBW/455). Public Health Act approval was obtained from each of the public hospitals permitting access to medical records without the need for informed consent.

### Sample size estimation

Based on multiple regression modelling, it had been calculated that at least 192 patient cases for each condition were required to achieve a power of 80% with 5% level of significance (effect size 0.10), accounting for the number of potential predictor variables included for each condition (27 KOA, 27 SIS, 29 LBP). This calculation also accounted for approximately 10% missing data.

### Criteria for a poor response to outcome (dependent variable)

A poor response to management for each patient case was determined by evaluating if they had achieved a minimal clinically important difference (MCID) based on the change scores of the region-specific disability questionnaires between the initial consultation and discharge. The MCID scores were used as the intent of the study was to specifically identify prognostic factors relevant to patients who have a poor response (ie. did not achieve MCID) from the studied intervention as they are the patients of the highest priority to identify clinically.

These region-specific disability questionnaires are routinely recorded pre (initial consultation) and post-intervention (at discharge from the service) and included the -.

1. Western Ontario and McMaster Universities Osteoarthritis Index (WOMAC) for the KOA group: This measure is used to evaluate the condition of patients with osteoarthritis of the knee and hip, and includes factors such as pain, stiffness, and physical functioning [28, 29]. In this study the WOMAC was scored using the relevant items from the Knee Injury and Osteoarthritis Outcome Score [30, 31] that is routinely measured for patients with KOA in the service, but could not be used due to the lack of published MCID. The MCID of the WOMAC has been reported as a reduction in score of 10 points or greater [32].
2. Shoulder Pain and Disability Index (SPADI) for the SIS group: This measure contains items for the domains of shoulder pain and disability (eg, self-care, lifting) and has a reported MCID of a 20 point reduction or greater [33].
3. Oswestry Disability Index (ODI) for the LBP group: This index is used to quantify disability for low back pain and contains items concerning pain intensity and function (eg. lifting, self-care) and has a MCID of a 10 point reduction or greater [34].

Change scores between the initial consultation and discharge from the service for each patient case were dichotomized (binary outcome) as either not achieving (reference outcome of a poor response) or achieving, the MCID for the relevant outcome measure.

### Potential predictor variables (independent variables)

Potential predictor variables were comprised of routine clinical information recorded at the patient’s initial consultation with the service team leader. This information included questionnaires and recorded information from the patient interview. These assessment items have progressively been selected over the development of the service by the team leaders to be clinically informative for the patient population. It was decided a priori that physical examination measures would not be collected for the purpose of this audit due to the known lack of standardisation for recording these items across the different sites and between team leaders. The included potential predictor variables (including units of measurement) are described below and in Table 1:

1. Demographic and general health information including age (years), gender (female/male), Body Mass Index (kilogram/meters^2^), comorbidities (number), and medications (number).
2. The Assessment of Quality of Life questionnaire (4 Dimension - utility measure score/1) which is a measure of health-related quality of life incorporating four domains (Independent Living, Mental Health, Relationships, Senses) [35].
3. Psychological questionnaires including the Depression, Anxiety and Stress Scale (DASS-21) that measures dimensions of depression (score/42), anxiety (score/42), and stress (score/42) [36], and the Pain Self-Efficacy Questionnaire (score/60). This assesses the confidence of people with ongoing pain in performing activities (eg. household chores, socialising, work) while in pain and is related to measures of pain-related disability, coping strategies, concordance to management programs, and functional outcomes [37].
4. Patient reported symptom characteristics including symptom severity (Pain Visual Analogue Scale Score/100 mm) [38], symptom distribution (extracted from medical record body chart that matched predetermined potential symptom regions as listed for each condition in Table 1), symptom duration (months), and mechanisms of onset (traumatic onset/not traumatic) of the condition.
5. Functional deficits as evaluated by the Patient Specific Functional Scale (score/10) that has been shown to be valid and responsive measure of function in patients with musculoskeletal disorders [39].
6. Prior management pathway for the condition including any previous consultations with orthopaedic medical specialists (yes/no), or previous surgery (yes/no) for the same condition.
7. Any documented radiological findings specifically investigating the patient’s condition matching predetermined potential radiological findings, as listed for each condition in Table 1.

**Table 1:** Group means (± standard deviation) for the variables tested for their relationship with not achieving a minimal clinically important difference (MCID) in outcome in response to the non-surgical multidisciplinary management of KOA, SIS, and LBP

Variables	Knee Osteoarthritis (*n* = 190)	Shoulder Impingement Syndrome (*n* = 199)	Low Back Pain (*n* = 242)
Age (years)	59.61 ± 10.48	60.75 ± 12.95	51.52 ± 15.02
Gender (% male)	39%*	45%*	44%
Body Mass Index (kilogram/meters^2^)	33.23 ± 7.29	30.51 ± 7.41	30.87 ± 7.73
Comorbidities (number)	3.26 ± 2.17	3.03 ± 2.24*	2.53 ± 2.17
Medications (number)	4.86 ± 3.6	5.22 ± 4.59	4.54 ± 4.41
Quality of Life (utility score/1)	0.5 ± 0.26*	0.53 ± 0.26	0.44 ± 0.26
Depression, Anxiety and Stress Scale			
Depression (score/42)	8.35 ± 10.21*	7.68 ± 9.92	10.45 ± 10.19
Anxiety Score (score/42)	6.23 ± 7.42*	5.74 ± 7.65	9.01 ± 8.76
Stress Score (score/42)	9.92 ± 9.53*	10.1 ± 10.15	13.83 ± 10.47
Pain Self-Efficacy (score/60)	34.89 ± 14.88*	37.04 ± 16.01	31.29 ± 14.87*
Pain Severity (score/100 mm)	56.36 ± 21.94	57.67 ± 23.5	58.03 ± 20.99
Function (score/10)	3.81 ± 1.71*	3.41 ± 1.96	3.85 ± 1.52
Symptom Duration (months)	61.09 ± 82.25	24.89 ± 57.4	93.19 ± 112.61*
Traumatic Onset (% traumatic, (n))	17% (32)	29% (57)	12% (30)
Previous Consultation (% yes, (n))	13% (24)	4% (8)	12% (28)*
Previous Surgery (% yes, (n))	13% (24)	0.5% (1)	5% (12)
Symptom Distribution (% cases, n) as recorded in the medical record body chart: *Knee Osteoarthritis* – Pain reported; lower back/pelvic/hip region (40.53%, 77), lower leg (12%, 23), bilateral knee (40%, 76)*. *Shoulder Impingement Syndrome* – Pain reported; cervical (27%, 53), acromioclavicular (16%, 32), forearm (29%, 58), bilateral shoulder (16%, 31). *Low Back Pain* - Pain reported; bilateral lower back 74% (178), one leg 47% (114)*, both legs 21% (51), thoracic and/or cervical region 16% (39)*. Paraesthesia and/or anaesthesia reported in the legs 41% (99).			
Radiological Findings (% cases, n) as reported in medical records: *Knee Osteoarthritis* – Medial (89%, 169), lateral (45%, 86) or patella (64% (122) compartment osteoarthritis, or tri-compartmental osteoarthritis (30%, 56). Osteoarthritis rated as severe (42%, 80)*. Reported meniscus pathology (25%, 47), valgus (9%, 18) or varus (18% 35) alignment. *Shoulder Impingement Syndrome* – Glenohumeral (19%, 37) or acromioclavicular (51%, 101) joint osteoarthritis, subacromial bursa distension (53%, 106), supraspinatus (67%, 134), infraspinatus (5%, 10)*, or subscapularis (15%, 30) muscle tendon tears. Multiple rotator cuff tendon tears; 1 (53%, 105), 2 (14%, 28), 3 (3%, 5). *Low Back Pain* – Lumbar degenerative changes (71%, 171), disc pathology (63%, 153), vertebral body pathology (18%, 44), zygopophyseal joint pathology (29%, 70), neural compression (43%, 103)*, spondylolisthesis (18%, 44), canal stenosis (31%, 75), foraminal stenosis (24%, 59).			

*denotes variables with a univariate relationship (*p* ≤ 0.1) with a non-MCID in outcome. Further details regarding some variables are provided in the footnote

### Audit and data extraction

**Fig. 1:**
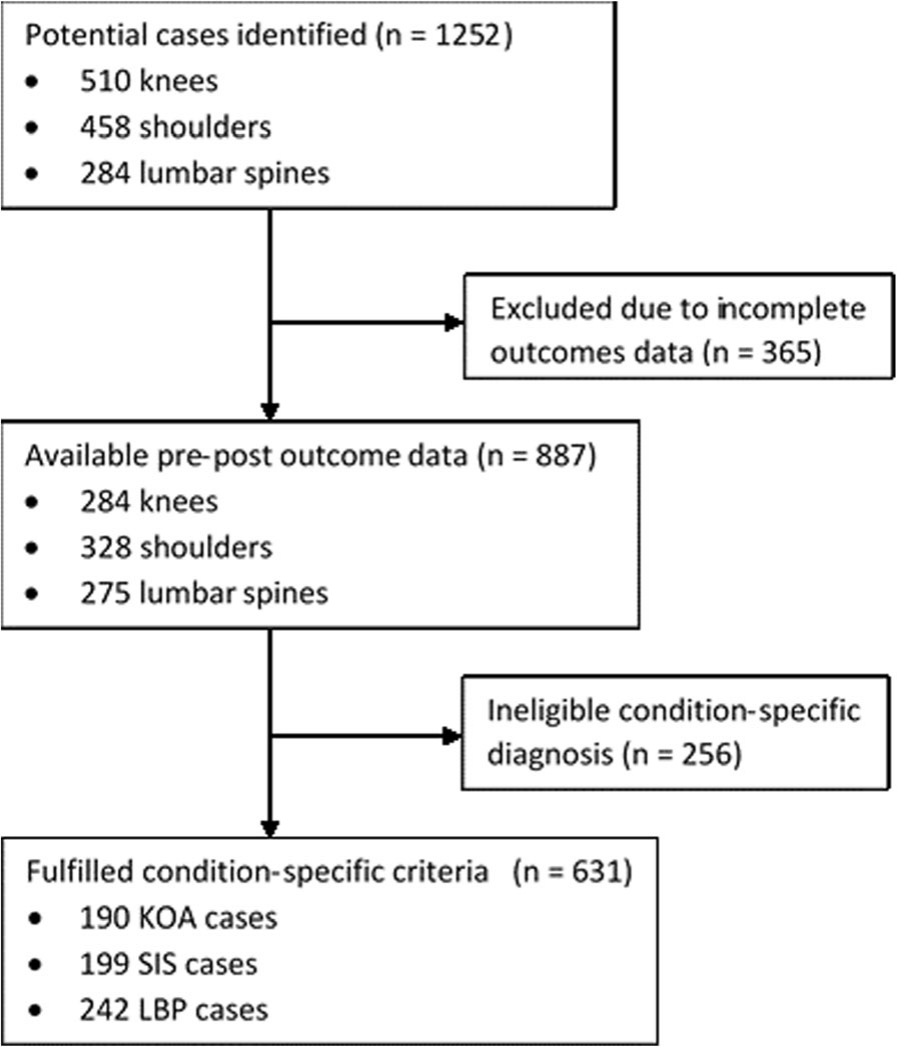
Flow diagram of the audit process

Auditing was undertaken by a single investigator (M.C) and the process is depicted in Fig. 1. Potential cases were initially identified from the service database according to body region managed (*n* = 1252; 510 knees, 284 lumbar, 458 shoulders) and their medical records reviewed. Only cases that included both pre- and post-intervention region-specific questionnaire scores were included (*n* = 887; 284 knees, 328 shoulders, 275 lumbar). Further cases were then excluded if they did not meet the eligibility criteria for the three orthopaedic conditions resulting in a total of 631 eligible cases (KOA, *n* = 190; SIS, *n* = 199; LBP, *n* = 242).

The inclusion criteria for the conditions included; 1/persistent knee pain in addition to radiologically identified knee osteoarthritis for the KOA group, 2/ reported pain in the lumbar/buttock region with or without leg symptoms for the LBP group, and 3/patients in the SIS group had to have been diagnosed with sub-acromial impingement syndrome and/or rotator cuff disease, with no indication of adhesive capsulitis, acromioclavicular joint injury, or recent or recurrent glenohumeral joint dislocation/subluxation/fracture. For all three conditions, cases had been naturally excluded by the standard service screening process, which excluded those presenting with potentially serious medical conditions requiring urgent referral to the medical specialist, or those not consenting to undertake non-surgical rehabilitation.

### Statistical analysis

All statistical analysis was performed using SPSS (version 20). The analysis was conducted separately for each of the musculoskeletal conditions (KOA, SIS, LBP) but followed an identical approach. Firstly the potential predictor variables (ie. patient demographics, quality of life and psychological measures, symptom characteristics, functional measure, prior management, radiological findings) were tested for a univariate relationship with the clinical management outcome of interest (ie. non- MCID of either the WOMAC, SPADI, or ODI) using independent-samples t-tests for continuous variables, and chi-square tests for categorical variables. Variables with a univariate relationship with the clinical outcome of a significance level of *p* ≤ 0.1 were retained as potential prediction variables [40]. Potential predictor variables were entered into a base binary logistic regression model which commenced with a full model, sequentially removing the most insignificant variables until all remaining were significant. This process was repeated for all three conditions separately to determine the most accurate set of variables for the prediction of a non-MCID outcome. Odds Ratios (OR) and 95% confidence intervals for significant variables were calculated. Standardised residuals of the fitted models were examined for outliers and no outliers were identified in each of the three models.

## Results

At baseline the region-specific disability questionnaire scores (± standard deviation) were 51.94% (18.24%) for the WOMAC (KOA group), 59.29% (21.8%) for the SPADI (SIS group), and 40.24% (15.31%) for the ODI (LBP group). Overall 98/190 (51.58%) of patients with KOA, 93/199 (46.73%) of patients with SIS, and 120/242 (49.59%) of patients with LBP, fit the criteria as a non-responder (ie. not achieving a MCID). The patient characteristics showing a univariate relationship with a non-MCID outcome in response to non-surgical management of KOA, SIS, and LBP are shown in Table 1.

Following logistic regression analysis, factors predictive of a non-MCID outcome included higher levels of anxiety (OR 1.11, 95% CI 1.03–1.21, *p* < 0.02) and lower functional score (OR 0.76, 95% CI 0.59–0.97, *p* < 0.03) for KOA, higher number of comorbidities (OR 1.16, 95% CI 1.02–1.32, *p* < 0.03) for SIS, and coexisting cervical or thorax pain (OR 2.1, 95% CI 1.02–4.4, *p* = 0.04) and lower pain self-efficacy (OR 0.98, 95% CI 0.96–1, *p* = 0.02) for LBP (Table 2). A Hosmer and Lemeshow goodness of fit test was conducted for all estimated models, and their insignificance (*p* > 0.28) suggest that the estimated models were a good fit to the sample data.

**Table 2:** Patient characteristics demonstrating a relationship with not achieving a minimal clinically important difference (MCID) following the non-surgical management of KOA, SIS, and LBP

Condition	No MCID Mean ± Standard Deviation or % (n)	MCID (Reference) Mean ± Standard Deviation or % (n)	Adjusted OR (95% Confidence Intervals)
Low Back Pain			
Cervical and/or thoracic pain			
Present	66.7% (26)	33.3% (13)	2.1 (1.02–4.4)^A^
Absent	46.3% (94)	53.7% (109)	
Pain Self Efficacy Score	28.82 ± 13.81 (120)	33.72 ± 15.51 (122)	0.98 (0.96–1)^A^
Shoulder Impingement Syndrome			
Comorbidities	3.55 ± 2.31 (133)	2.77 ± 2.28 (124)	1.16 (1.02–1.32)^A^
Knee Osteoarthritis			
Psychological Anxiety	7.89 ± 8.91 (91)	4.54 ± 5.02 (89)	1.11 (1.03–1.21)^A^
Patient Specific Functional Scale	3.37 ± 1.38 (53)	4.19 ± 1.88 (62)	0.76 (0.59–0.97)^A^

Significant at ^A^*P* < 0.05

## Discussion

The findings of this study indicate that general health factors potentially present some barrier to effective non-surgical multidisciplinary rehabilitation of common orthopaedic conditions managed in the tertiary care setting. The findings share both similarities and discrepancies with previous literature, albeit many of the previous studies performed in primary care settings. For example the psychological factors observed to be predictive of a poor response in KOA (higher levels of anxiety) and LBP (lower pain self-efficacy) are consistent with those previously reported for LBP (catastrophizing, depressed mood) [19] and shoulder disorders (patient expectations, self-efficacy) [16]. Similarly higher comorbidity has been observed as a risk factor for poorer outcome in shoulder disorders in this current study and in previous studies [16, 18]. In general findings are also consistent with factors identified in a systematic review to be generic prognostic indicators of poor recovery such as widespread pain, higher disability, and comorbid psychological factors [14].

Interestingly only one variable specific to the condition (low functional capacity in KOA) in this study was predictive of a poor response. No other condition-specific measures including those relating to symptoms (severity, duration), mechanisms of onset, previous management, or radiological findings were found to be related to a poor response to rehabilitation. This is in contrast to previous studies that have shown condition-specific factors to be associated with poor response to rehabilitation for KOA (pain severity, ligament laxity, pain during physical examination) [15] and shoulder conditions (pain severity and duration [18, 41], subacromial injection response [3], acromion morphology [17]). It should be acknowledged however, that this discrepancy may reflect the limited condition-specific factors that were measured in this study, including the lack of physical assessment measures. Irrespective, the findings of this study have an important message for clinicians managing these disorders to consider the total patient presentation when considering prognostic indicators of recovery and not just factors directly related to the condition.

It should be noted that the strength of the relationship between some of the predictive variables and not achieving a MCID in outcome was relatively modest (OR range 0.76–2.1). For example the anxiety level of the KOA group not achieving a MCID (7.89 points) is at the lower end of the mild anxiety category (8–9 points) [36]. Irrespective, these patient characteristics observed prior to management suggests the presence of a more severe or complex condition (widespread pain, low functional capacity, comorbidity, low pain self-efficacy, higher anxiety) that may potentially affect the individual’s capacity to achieve a satisfactory benefit from non-surgical multidisciplinary management. Certainly the findings of this exploratory study warrants further investigation of these and other patient characteristics in their capacity to identify at risk patients. This is especially the case as predictive characteristics may be different between musculoskeletal conditions and between different health settings (primary versus tertiary).

### Limitations

There are limitations of this study due its retrospective methodology. These include unrecorded, missing, or illegible information within the medical records. Additionally other potentially useful variables such as physical examination findings were not included as they had not been recorded in a standardised manner across participating sites. Therefore, there may be other informative clinical variables not evaluated in this retrospective study which justifies further prospective investigation, with the aim of eventually developing effective screening tools to identify at risk patients. It should also be noted that there are limitations associated with dichotomising clinical outcomes (ie. not achieving/achieving a MCID). While MCID scores were purposefully used in this study to specifically identify prognostic factors relevant to patients who report a poor response, potentially regression modelling based on the original score may give better power in predictor selection and effect estimations. Despite these limitations, the use of measures collected in the clinical environment in this study, combined with the collection of data at seven tertiary hospital sites, strengthens the potential clinical meaningfulness of the findings.

## Conclusions

General health issues (eg. coexisting conditions, psychological factors) are potential barriers to effective non-surgical multidisciplinary rehabilitation of common orthopaedic conditions in tertiary care. Specifically the findings suggest that factors other than just the biological severity of the orthopaedic condition may impact the patient’s response to rehabilitation. From a clinical perspective, the early identification of prognostic indicators of a poor response in an individual patient should be considered within the context of management planning for that patient.

## Abbreviations

KOA: Knee Osteoarthritis
LBP: Low Back Pain
MCID: Minimal Clinically Important Difference
ODI: Oswestry Disability Index
OR: Odds Ratio
SIS: Shoulder Impingement Syndrome
SPADI: Shoulder Pain and Disability Index
WOMAC: Western Ontario and McMaster Universities Osteoarthritis Index
